# Evaluation of congruence among dietary supplement use and motivation for supplementation in young, Canadian athletes

**DOI:** 10.1186/s12970-015-0110-y

**Published:** 2015-12-16

**Authors:** Jill A. Parnell, Kristin Wiens, Kelly Anne Erdman

**Affiliations:** Department of Physical Education and Recreation Studies, Mount Royal University, 4825 Mount Royal Gate SW, Calgary, Alberta T3E 6K6 Canada; Department of Behavioral Health and Nutrition, University of Delaware, 026 North College Avenue, Newark, Delaware 19716 USA; Sport Medicine Centre, University of Calgary, 2500 University Drive NW, Calgary, Alberta T2N 1N4 Canada

**Keywords:** Sport supplements, Dietary ergogenic aids, Supplementation rationale, Dietary supplements

## Abstract

**Background:**

Dietary supplement use is endemic in young athletes; however, it is unclear if their choices are congruent with their motivation for supplementation and the established benefits of the dietary supplements. The aim of this study was to evaluate the relationships between dietary supplement use and self-reported rationale in young athletes.

**Methods:**

Canadian athletes (*n* = 567; 11–25 years; 76 % club or provincial level, 24 % national or higher) completed a questionnaire designed to assess supplementation patterns and motivation for supplementation. Chi square tests examined associations between dietary supplements and self-reported rationale for use.

**Results:**

Vitamin and mineral supplements, including vitamin-enriched water, were associated with several health- and performance- related reasons (*p* < 0.001). Branched chain amino acids (BCAA) and glutamine were linked to improving diet and immune function (*p* < 0.01), but were more strongly associated with performance reasons, as were performance foods (protein powder, sport bars, sport gels, etc.). Plant extracts and fatty acids were primarily associated with health reasons, particularly immune support (*p* < 0.001).

**Conclusions:**

Congruencies exist between performance rationales and supplementation for common ergogenic aids, however, less so for vitamin and mineral supplements, vitamin-enriched water, and plant extracts. Incongruences were found between fatty acids, protein supplements, vitamin and mineral supplements, vitamin-enriched water, and plant extracts and health motivators for supplementation. Educational interventions are essential to ensure young athletes are using dietary supplements safely and effectively.

## Background

Athletes experience significant internal and external pressures inspiring them to set high performance goals. Young athletes have the added requirement of supporting growth and promoting healthy maturation, which can be especially difficult in sports encouraging low body weight or body fat. Further, challenges identified as unique to young athletes include: decreased metabolic efficiency (increased energy requirements per kilogram of body weight as compared to adults performing the same type of exercise), preferential fat oxidation, and reduced ability to thermoregulate while exercising [1].

In order to maximize performance, athletes often turn to supplements in the belief they will help them stay competitive and healthy. Petroczi et al. [2] report 78 % of young athletes thought nutritional supplementation was not essential for athlete success; however, note the contradiction in actual habits, where 48 % of these same athletes were using nutritional supplements. In this case, the most common reasons for supplement use included “maintaining strength”, “avoiding sickness” and “endurance enhancement”. Recent work in young, Canadian athletes found the top five reasons for supplement use were “stay healthy”, “increase energy”, “immune system”, “recovery” and “overall athletic performance” [3].

Supplementation is common in young athletes with prevalence estimates varying depending on the specific population and dietary supplements included in the assessment. We report one of the highest rates of supplement use at 98 % in young, Canadian athletes [3]. The reason for higher rates in young, Canadian athletes is unclear; however, is likely due to the broad definition of dietary supplements based on the Dietary Supplement Health and Education Act in the United States [4] and the Canadian Natural Health Products Regulations [5]. Popular supplements consumed by young athletes include, but are not restricted to: sport drinks (i.e., carbohydrate electrolyte solutions), energy drinks (i.e., caffeinated beverages), vitamin-enriched water, vitamin or minerals either individually or in combination, sport bars, protein powders, creatine, fatty acid preparations (i.e. omega-3 supplements, fish oil, flax seed oil, cod liver oil), and plant extracts [2, 3, 6].

Dietary supplement patterns and motivations for supplement use in young athletes have been established. What remains to be determined is if there is a link between the purported functions of the dietary supplements athletes are consuming and their self-reported rationale(s) for supplement use. One study, among youth, reports perceived associations between energy drinks and enhanced endurance, as well as maintaining strength and creatine and whey protein [2]. Conversely, overall there was significant incongruence between rationale for supplement use and actual practice in these athletes [2]. The same group reported a high degree of incongruence between supplementation and performance-related rationales in athletes who were primarily between 19 and 29 years of age [7]. Notable exceptions include the associations between creatine, whey protein and strength maintenance, as well as creatine and the ability to train longer. Conversely, the authors single out iron, vitamin C and ginseng as particularly problematic when it comes to the athletes’ perception of the potential performance benefits of these supplements as compared to their actual benefits [7].

The aim of the present study was to assess the relationship between supplementation and self-reported rationale for supplement use with the goal of developing a better understanding of the knowledge and practices of young athletes. There is currently very little information available in this area, and even less so in developing, Canadian athletes competing primarily at lower performance levels. The focus on club and provincial level athletes is crucial, as this is the extent at which most youth compete. This research will contribute to the field, as it will identify knowledge gaps that can be addressed by athlete support personnel.

## Methods

### Participants

Athletes were recruited from the province of Alberta representing a variety of sporting organizations. All athletes were between the ages of 11 and 25 years and participated in structured training for a minimum of five hours per week. Athletes from all levels of competition were included, however, 76 % competed at the provincial level or lower and 24 % competed nationally or internationally. Power calculations using a prior study in high-performance, Canadian athletes and a margin of error (4 % with 95 % confidence and a power of 80 %) established that 567 participants were required [6]. All athletes provided written consent or written assent with written consent from a parent or guardian if they were under the age of 18 years. The study was approved by the Mount Royal University Human Research Ethics Board.

### Study design

The athletes completed a paper version of a content validated and reliability tested questionnaire. Detailed information on the questionnaire development and administration has been published previously [3]. Briefly, a panel of experts reviewed all questions for content and clarity and their suggestions were incorporated into a revised version. A second panel of experts reviewed the revised version and all questions were rated using a Likert scale. Test re-test data was collected from 31 participants and analyzed by weighted kappa coefficients to assess reliability. The questionnaire was administered in person by the study investigators. The participants were free to ask the researchers for additional information or to clarify the questions; however, subjects completed the questionnaire without assistance from parents, guardians, coaches, trainers, etc. Further details regarding the administration of the questionnaire are available [3]. The questionnaire asked for information regarding their use of dietary supplements, reason(s) for taking supplements, and sources of information. The questions analyzed here include: 1) “Do you know the kinds of supplements you take? (Do you know what the supplements you take are called or does someone else give you your supplements?)” with “I’m not sure which supplements I take”, “I know some of the supplements I take”, “I know many of the supplements I take”, and “I know all of the supplements I take” as response options. 2) “Do you know why you take each supplement? (Do you know what each supplement is for or do you take the supplement and not question it?)” with “I’m not sure why I take each supplement”, “I know why I take some of my supplements”, “I know why I take many of my supplements”, and “I know why I take all of my supplements” as response options. 3) “How often have you taken the following dietary supplements in the last 3 months? Please circle Daily, Weekly, Monthly, If I’m Sick or Never in the “How Often” column. If you do take a supplement, please write the exact one in the “Supplement Name” column if you know it”. Options for supplements grouped by category can be found in Table 1. 4) “What are your reason(s) for taking dietary supplements? Please check all that apply”. Options for reasons for supplement use grouped by category can be found in Table 2. Athletes were not specifically asked to provide a rationale for each supplement they consumed. Relevant information including age, gender, sport type, level of competition, and average number of training hours per week was also collected.

**Table 1:** Supplements listed on the questionnaire

Supplement Category	Individual Supplements Listed
Dietary/Medical	Multi-vitamin/Multi-mineral Vitamin C B-Vitamins Vitamin D Vitamin E Vitamin-enriched water Calcium Iron Magnesium
Other Supplements	Fatty acid preparations Plant/herbal extracts
Ergogenic Aids (Muscle Building/Increased Energy)	Branched chain amino acids Beta-alanine Glutamine Creatine Energy drinks
Performance Foods	Protein powder Sport/electrolyte drinks Recovery drinks Protein/sport bar Sport gels Gummy/bean

**Table 2:** Reasons for supplementation listed on the questionnaire

Reason Category	Individual Reasons Listed
Health-Related	Medical (your doctor told you to) To improve your diet (food you eat everyday) Stay healthy Enhance immune system (so you don’t get sick)
Performance-Related	Increase or maintain muscle mass, strength, and/or power Increase endurance (how long you can exercise) Increase energy (so you don’t feel tired) Enhance overall athletic performance Improve exercise recovery (after exercise)
Influence of Others	Because others (friends, family, teammates) do Someone told you to (coach, parent, friend etc.)

### Criteria for evaluating outcome measures

Athletes were categorized by gender, age group (11–13 years, 14–16 years, 17–18 years, and 19–25 years), sport classification (power and strength, endurance, intermittent, and aesthetic), and level of competition (club, provincial, national or higher), as previously described [3]. Rationales for supplementation were grouped into health-related reasons, performance-related reasons, or influence-of-others.

### Statistical analyses

Intake for each supplement was categorized into ‘Yes or No’ based on any reported use. Chi squared tests were used to determine congruency between individual supplements and self-reported rationale for supplement use overall and between gender, age, sport type, and competition level groups. Cramer’s V was calculated to describe the magnitude of association between variables and 0.10 was considered a small effect size, 0.30 a medium effect size and 0.50 a large effect size [8]. All analyses were conducted in SPSS Version 22.0 (Armonk, NY: IBM Corp).

## Results

Three hundred and thirty five female athletes and 232 male athletes completed the questionnaire. One questionnaire was omitted from the analysis as the respondent reported consuming all supplements on a daily basis. Detailed information regarding the athletes’ ages, gender, sport types, and competition levels have been published previously [3].

### Self-evaluation of knowledge for reasons for supplementation

When athletes were asked to evaluate their knowledge of the reasons for taking dietary supplements six percent were “not sure”, 22 % said they knew the reasons for “some” of the supplements, 30 % said they knew the purpose of “many” of the supplements they took, and 42 % reported they knew the reasons for taking “all” of their supplements. There were significant differences within age groups (*p* = 0.01; V = 115) with 43 % of those between the ages of 11 and 13 years claiming they knew the purpose of all of their supplements as compared to 60 % of those in the 19–25 age group. No differences were noted in self-evaluated knowledge of the reasons for taking supplements within the gender, sport type, or level of competition groupings.

### Supplement choice and health related reasons

Eighty-eight percent of athletes reported at least one health-related reason for supplementation. Vitamin and mineral supplements, either individual or in combinations, were closely associated with all health-related reasons (Table 3). Conversely, vitamin-enriched water was associated with “improve diet” and “stay healthy” but the associations were not as strong as with traditional forms of vitamin and mineral supplements.

**Table 3:** Associations between supplement use and health-related reasons for supplementation

Supplement	Medical (19 %)	Improve Diet (43 %)	Stay Healthy (81 %)	Support Immune System (52 %)
Multi-vitamin/Multi-mineral (67 %)	***χ*** ^**2**^ **= 12.576 ** ***p*** **< 0.001** **V = 0.150**	*χ* ^2^ = 7.057 *p* = 0.008 V = 0.112	***χ*** ^**2**^ **= 32.679 ** ***p*** **< 0.001** **V = 0.241**	***χ*** ^**2**^ **= 41.807 ** ***p*** **< 0.001** **V = 0.273**
Vitamin C (66 %)	*χ* ^2^ = 5.825 *p* = 0.016 V = 0.102	***χ*** ^**2**^ **= 15.603 ** ***p*** **< 0.001** **V = 0.166**	***χ*** ^**2**^ **= 35.707 ** ***p*** **< 0.001** **V = 0.252**	***χ*** ^**2**^ **= 32.878 ** ***p*** **< 0.001** **V = 0.241**
B Vitamins (32 %)	*χ* ^2^ = 3.623 *p* = 0.057 V = 0.080	***χ*** ^**2**^ **= 12.740 ** ***p*** **< 0.001** **V = 0.150**	***χ*** ^**2**^ **= 14.195 ** ***p*** **< 0.001** **V = 0.159**	***χ*** ^**2**^ **= 17.709 ** ***p*** **< 0.001** **V = 0.177**
Vitamin E (26 %)	*χ* ^2^ = 2.136 *p* = 0.144 V = 0.062	*χ* ^2^ = 8.109 *p* = 0.004 V = 0.120	*χ* ^2^ = 8.428 *p* = 0.004 V = 0.122	***χ*** ^**2**^ **= 10.368 ** ***p*** **= 0.001** **V = 0.136**
Vitamin D (48 %)	***χ*** ^**2**^ **= 15.691 ** ***p*** **< 0.001** **V = 0.167**	***χ*** ^**2**^ **= 12.615 ** ***p*** **< 0.001** **V = 0.150**	***χ*** ^**2**^ **= 32.496 ** ***p*** **< 0.001** **V = 0.240**	***χ*** ^**2**^ **= 22.208 ** ***p*** **< 0.001** **V = 0.199**
Vitamin-enriched Water (65 %)	*χ* ^2^ = 3.386 *p* = 0.066 V = 0.077	*χ* ^2^ = 4.664 *p* = 0.031 V = 0.091	*χ* ^2^ = 5.183 *p* = 0.023 V = 0.096	*χ* ^2^ = 2.510 *p* = 0.113 V = 0.067
Iron (27 %)	***χ*** ^**2**^ **= 17.142 ** ***p*** **< 0.001** **V = 0.174**	***χ*** ^**2**^ **= 20.320 ** ***p*** **< 0.001** **V = 0.190**	***χ*** ^**2**^ **= 13.759 ** ***p*** **< 0.001** **V = 0.156**	***χ*** ^**2**^ **= 16.803 ** ***p*** **< 0.001** **V = 0.172**
Calcium (43 %)	***χ*** ^**2**^ **= 22.947 ** ***p*** **< 0.001** **V = 0.202**	***χ*** ^**2**^ **= 30.458 ** ***p*** **< 0.001** **V = 0.232**	***χ*** ^**2**^ **= 31.370 ** ***p*** **< 0.001** **V = 0.236**	***χ*** ^**2**^ **= 26.782 ** ***p*** **< 0.001** **V = 0.218**
Magnesium (17 %)	*χ* ^2^ = 1.001 *p* = 0.317 V = 0.042	*χ* ^2^ = 9.440 *p* = 0.002 V = 0.129	*χ* ^2^ = 8.538 *p* = 0.003 V = 0.123	***χ*** ^**2**^ **= 12.815 ** ***p*** **< 0.001** **V = 0.151**
Protein Powder (51 %)	*χ* ^2^ = 1.692 *p* = 0.193 V = 0.055	***χ*** ^**2**^ **= 14.825 ** ***p*** **< 0.001** **V = 0.162**	*χ* ^2^ = 5.085 *p* = 0.024 V = 0.095	***χ*** ^**2**^ **= 16.882 ** ***p*** **< 0.001** **V = 0.173**
BCAA (8 %)	*χ* ^2^ = 0.233 *p* = 0.629 V = 0.020	*χ* ^2^ = 9.765 *p* = 0.002 V = 0.132	*χ* ^2^ = 2.415 *p* = 0.120 V = 0.065	*χ* ^2^ = 7.317 *p* = 0.007 V = 0.114
Beta-alanine (4 %)	*χ* ^2^ = 5.191 *p* = 0.023 V = 0.096	*χ* ^2^ = 3.369 *p* = 0.066 V = 0.077	*χ* ^2^ = 0.871 *p* = 0.351 V = 0.039	*χ* ^2^ = 0.228 *p* = 0.633 V = 0.020
Glutamine (8 %)	*χ* ^2^ = 0.260 *p* = 0.610 V = 0.021	***χ*** ^**2**^ **= 13.015 ** ***p*** **< 0.001** **V= 0.152**	*χ* ^2^ = 4.687 *p* = 0.030 V = 0.091	*χ* ^2^ = 6.923 *p* = 0.009 V = 0.111
Fatty Acids (40 %)	***χ*** ^**2**^ **= 13.960 ** ***p*** **< 0.001** **V = 0.157**	*χ* ^2^ = 9.602 *p* = 0.002 V = 0.130	***χ*** ^**2**^ **= 21.589 ** ***p*** **< 0.001** **V = 0.196**	***χ*** ^**2**^ **= 34.971 ** ***p*** **< 0.001** **V = 0.249**
Energy Drinks (27 %)	*χ* ^2^ = 4.642 *p* = 0.031 V = 0.091	*χ* ^2^ = 8.362 *p* = 0.004 V = 0.122	*χ* ^2^ = 0.757 *p* = 0.384 V = 0.037	*χ* ^2^ = 0.285 *p* = 0.593 V = 0.022
Sport/Electrolyte Drinks (90 %)	*χ* ^2^ = 0.817 *p* = 0.366 V = 0.038	*χ* ^2^ = 1.164 *p* = 0.281 V = 0.045	*χ* ^2^ = 5.081 *p* = 0.024 V = 0.095	*χ* ^2^ = 1.172 *p* = 0.279 V = 0.046
Sport Gels/Gummies (26 %)	*χ* ^2^ = 1.315 *p* = 0.251 V = 0.048	*χ* ^2^ = 0.555 *p* = 0.456 V = 0.031	*χ* ^2^ = 5.810 *p* = 0.016 V = 0.101	*χ* ^2^ = 3.462 *p* = 0.063 V = 0.078
Sport/Protein Bar (71 %)	*χ* ^2^ = 0.961 *p* = 0.327 V = 0.041	***χ*** ^**2**^ **= 10.708 ** ***p*** **= 0.001** **V = 0.138**	***χ*** ^**2**^ **= 11.233 ** ***p*** **= 0.001** **V = 0.141**	***χ*** ^**2**^ **= 10.456 ** ***p*** **= 0.001** **V = 0.136**
Recovery Drinks (31 %)	*χ* ^2^ = 0.166 *p* = 0.684 V = 0.017	***χ*** ^**2**^ **= 10.532 ** ***p*** **= 0.001** **V = 0.137**	*χ* ^2^ = 6.735 *p* = 0.009 V = 0.109	*χ* ^2^ = 9.528 *p* = 0.002 V = 0.130
Creatine (7 %)	*χ* ^2^ = 0.015 *p* = 0.903 V = 0.005	*χ* ^2^ = 1.416 *p* = 0.234 V = 0.050	*χ* ^2^ = 0.123 *p* = 0.726 V = 0.015	*χ* ^2^ = 1.617 *p* = 0.204 V = 0.054
Plant Extracts (47 %)	*χ* ^2^ = 9.536 *p* = 0.002 V = 0.130	*χ* ^2^ = 7.859 *p* = 0.005 V = 0.118	*χ* ^2^ = 9.864 *p* = 0.002 V = 0.132	***χ*** ^**2**^ **= 49.130 ** ***p*** **< 0.001** **V = 0.295**

Statistically significant associations at *p* ≤ 0.001 are in bold. Percentages after the supplement type represent the percent of total athletes consuming the supplements listed. Percentages after the health-related reasons represent the percent of total athletes supplementing for the reasons listed

Athletes who consumed recovery drinks, sport/protein bars, protein powders, glutamine, and branched chain amino acids (BCAA) were also interested in improving their diet and supporting their immune system. Fatty acid supplementation was linked to all health-related reasons (Table 3).

Creatine, sport drinks, energy drinks, and sport gels and gummies were rarely associated with health-related reasons. Conversely, plant extracts were related to all health reasons for supplementation (Table 3). Plant extracts athletes consumed included: oil of oregano, ginseng, green tea pills, garlic, herbs, St John’s Wart, echinacea, and Cold FX (™).

Age did not have an effect on health-related reasons for choosing supplementation as the percentage of athletes choosing supplements for health-related reasons ranged from 87 to 89 % in the various age groups (*p* = 0.94; V = 0.026). Gender, level of competition or sport type did not impact health reasons for supplement choice.

### Supplement choice and performance related reasons

In total, 81 % of athletes reported performance reasons as a rationale for supplementation. Generally, vitamin supplements were associated with all performance reasons with the exception of recovery. There were variations depending on the specific vitamin; however, the strongest associations were found with the B vitamins and vitamin E. Vitamin-enriched water was associated with all performance-related reasons for choosing supplements (Table 4). Minerals followed a similar trend with associations between iron, calcium, and magnesium and all performance-related reasons with the exception of recovery, for which only magnesium showed an association (Table 4).

**Table 4:** Associations between supplement use and performance-related reasons for supplementation

Supplement	Muscle Mass/Strength (38 %)	Endurance (31 %)	Increase Energy (55 %)	Overall Athletic Performance (49 %)	Recovery (49 %)
Multi-vitamin/Multi-mineral (67 %)	***χ*** ^**2**^ **= 11.965** ***p*** **= 0.001** **V = 0.146**	*χ* ^2^ = 8.469 *p* = 0.004 V = 0.123	*χ* ^2^ = 0.203 *p* = 0.652 V = 0.019	*χ* ^2^ = 6.370 *p* = 0.012 V = 0.106	*χ* ^2^ = 5.773 *p* = 0.016 V = 0.101
Vitamin C (66 %)	*χ* ^2^ = 7.688 *p* = 0.006 V = 0.117	*χ* ^2^ = 5.278 *p* = 0.022 V = 0.097	*χ* ^2^ = 2.580 *p* = 0.108 V = 0.068	*χ* ^2^ = 4.662 *p* = 0.031 V = 0.091	*χ* ^2^ = 3.046 p = 0.081 V = 0.073
B Vitamins (32 %)	***χ*** ^**2**^ **= 11.002** ***p*** **= 0.001** **V = 0.140**	***χ*** ^**2**^ **= 20.610** ***p*** **< 0.001** **V = 0.191**	*χ* ^2^ = 6.675 *p* = 0.010 V = 0.109	***χ*** ^**2**^ **= 11.867 ** ***p*** **= 0.001** **V = 0.145**	*χ* ^2^ = 1.487 *p* = 0.223 V = 0.051
Vitamin E (26 %)	***χ*** ^**2**^ **= 25.084** ***p*** **< 0.001** **V = 0.211**	***χ*** ^**2**^ **= 27.410** ***p*** **< 0.001** **V = 0.221**	***χ*** ^**2**^ **= 10.883** ***p*** **= 0.001** **V = 0.139**	***χ*** ^**2**^ **= 18.510 ** ***p*** **< 0.001** **V = 0.181**	*χ* ^2^ = 2.257 *p* = 0.133 V = 0.063
Vitamin D (48 %)	*χ* ^2^ = 9.013 *p* = 0.003 V = 0.127	***χ*** ^**2**^ **= 12.488** ***p*** **< 0.001** **V = 0.149**	*χ* ^2^ = 0.619 *p* = 0.431 V = 0.033	*χ* ^2^ = 2.629 *p* = 0.105 V = 0.068	*χ* ^2^ = 0.616 *p* = 0.433 V = 0.033
Vitamin- enriched Water (65 %)	*χ* ^2^ = 5.594 *p* = 0.018 V = 0.100	***χ*** ^**2**^ **= 10.919** ***p*** **= 0.001** **V = 0.139**	***χ*** ^**2**^ **= 13.372** ***p*** **< 0.001** **V = 0.154**	***χ*** ^**2**^ **= 17.511 ** ***p*** **< 0.001** **V = 0.176**	*χ* ^2^ = 4.535 *p* = 0.033 V = 0.090
Iron (27 %)	*χ* ^2^ = 6.234 *p* = 0.013 V = 0.105	*χ* ^2^ = 7.387 *p* = 0.007 V = 0.114	*χ* ^2^ = 6.574 *p* = 0.010 V = 0.108	*χ* ^2^ = 7.795 *p* = 0.005 V = 0.117	*χ* ^2^ = 0.223 *p* = 0.637 V = 0.020
Calcium (43 %)	***χ*** ^**2**^ **= 25.438** ***p*** **< 0.001** **V = 0.212**	***χ*** ^**2**^ **= 10.571** ***p*** **= 0.001** **V = 0.137**	*χ* ^2^ = 9.816 *p* = 0.002 V = 0.132	***χ*** ^**2**^ **= 10.204 ** ***p*** **= 0.001** **V = 0.134**	*χ* ^2^ = 3.097 *p* = 0.078 V = 0.074
Magnesium (17 %)	***χ*** ^**2**^ **= 11.060** ***p*** **= 0.001** **V = 0.140**	***χ*** ^**2**^ **= 16.037** ***p*** **< 0.001** **V = 0.169**	*χ* ^2^ = 7.193 *p* = 0.007 V = 0.113	*χ* ^2^ = 7.827 *p* = 0.005 V = 0.118	*χ* ^2^ = 5.510 *p* = 0.019 V = 0.099
Protein Powder (51 %)	***χ*** ^**2**^ **= 120.609** ***p*** **< 0.001** **V = 0.462**	***χ*** ^**2**^ **= 27.927** ***p*** **< 0.001** **V = 0.223**	***χ*** ^**2**^ **= 12.172** ***p*** **< 0.001** **V = 0.147**	***χ*** ^**2**^ **= 47.946 ** ***p*** **< 0.001** **V = 0.292**	***χ*** ^**2**^ **= 51.470 ** ***p*** **< 0.001** **V = 0.302**
BCAA (8 %)	***χ*** ^**2**^ **= 21.995** ***p*** **< 0.001** **V = 0.198**	***χ*** ^**2**^ **= 14.313** ***p*** **< 0.001** **V = 0.159**	*χ* ^2^ = 7.943 *p* = 0.005 V = 0.119	***χ*** ^**2**^ **= 16.010 ** ***p*** **< 0.001** **V = 0.169**	*χ* ^2^ = 3.392 *p* = 0.065 V = 0.078
Beta-alanine (4 %)	*χ* ^2^ = 3.440 *p* = 0.064 V = 0.078	*χ* ^2^ = 5.451 *p* = 0.020 V = 0.098	*χ* ^2^ = 3.095 *p* = 0.079 V = 0.074	*χ* ^2^ = 2.462 *p* = 0.117 V = 0.066	*χ* ^2^ = 0.536 *p* = 0.464 V = 0.031
Glutamine (8 %)	***χ*** ^**2**^ **= 26.895** ***p*** **< 0.001** **V = 0.218**	***χ*** ^**2**^ **= 27.131** ***p*** **< 0.001** **V = 0.219**	*χ* ^2^ = 9.594 *p* = 0.002 V = 0.130	***χ*** ^**2**^ **= 15.530 ** ***p*** **< 0.001** **V = 0.166**	***χ*** ^**2**^ **= 15.335 ** ***p*** **< 0.001** **V = 0.165**
Fatty Acids (40 %)	***χ*** ^**2**^ **= 12.534** ***p*** **< 0.001** **V = 0.149**	*χ* ^2^ = 4.866 *p* = 0.027 V = 0.093	*χ* ^2^ = 0.302 *p* = 0.583 V = 0.023	*χ* ^2^ = 2.087 *p* = 0.149 V = 0.061	*χ* ^2^ = 0.267 *p* = 0.606 V = 0.022
Energy Drinks (27 %)	***χ*** ^**2**^ **= 14.246** ***p*** **< 0.001** **V = 0.159**	*χ* ^2^ = 5.024 *p* = 0.025 V = 0.094	***χ*** ^**2**^ **= 22.360** ***p*** **< 0.001** **V = 0.199**	*χ* ^2^ = 0.653 *p* = 0.419 V = 0.034	*χ* ^2^ = 4.312 *p* = 0.038 V = 0.087
Sport/Electrolyte Drinks (90 %)	***χ*** ^**2**^ **= 17.637** ***p*** **< 0.001** **V = 0.177**	*χ* ^2^ = 9.922 *p* = 0.002 V = 0.133	***χ*** ^**2**^ **= 19.795** ***p*** **< 0.001** **V = 0.187**	***χ*** ^**2**^ **= 16.349 ** ***p*** **< 0.001** **V = 0.170**	***χ*** ^**2**^ **= 18.945 ** ***p*** **< 0.001** **V = 0.183**
Sport Gels/Gummies (26 %)	***χ*** ^**2**^ **= 14.132** ***p*** **< 0.001** **V = 0.158**	*χ* ^2^ = 8.300 *p* = 0.004 V = 0.121	***χ*** ^**2**^ **= 12.120 ** ***p*** **< 0.001** **V = 0.146**	*χ* ^2^ = 8.098 *p* = 0.004 V = 0.120	***χ*** ^**2**^ **= 18.349 ** ***p*** **< 0.001** **V = 0.180**
Sport/Protein Bar (71 %)	***χ*** ^**2**^ **= 27.780** ***p*** **< 0.001** **V = 0.222**	***χ*** ^**2**^ **= 29.989** ***p*** **< 0.001** **V = 0.230**	***χ*** ^**2**^ **= 16.794** *** p*** **< 0.001** **V = 0.172**	***χ*** ^**2**^ **= 28.890 ** ***p*** **< 0.001** **V = 0.226**	***χ*** ^**2**^ **= 38.043 ** ***p*** **< 0.001** **V = 0.259**
Recovery Drinks (31 %)	***χ*** ^**2**^ **= 57.268** ***p*** **< 0.001** **V = 0.319**	***χ*** ^**2**^ **= 41.358** ***p*** **< 0.001** **V = 0.271**	***χ*** ^**2**^ **= 16.384 ** ***p*** **< 0.001** **V = 0.171**	***χ*** ^**2**^ **= 41.349 ** ***p*** **< 0.001** **V = 0.271**	***χ*** ^**2**^ **= 61.395 ** ***p*** **< 0.001** **V = 0.330**
Creatine (7 %)	***χ*** ^**2**^ **= 19.434** ***p*** **< 0.001** **V = 0.186**	***χ*** ^**2**^ **= 15.634** ***p*** **< 0.001** **V = 0.166**	*χ* ^2^ = 3.173 *p* = 0.075 V = 0.075	***χ*** ^**2**^ **= 10.546 ** ***p*** **< 0.001** **V = 0.137**	*χ* ^2^ = 6.629 *p* = 0.010 V = 0.108
Plant Extracts (47 %)	*χ* ^2^ = 8.426 *p* = 0.004 V = 0.122	*χ* ^2^ = 2.079 *p* = 0.149 V = 0.061	*χ* ^2^ = 7.471 *p* = 0.006 V = 0.115	*χ* ^2^ = 5.633 *p* = 0.018 V = 0.100	*χ* ^2^ = 3.194 *p* = 0.074 V = 0.075

Statistically significant associations at *p* ≤ 0.001 are in bold. Percentages after the supplement type represent the percent of total athletes consuming the supplements listed. Percentages after the performance-related reasons represent the percent of total athletes supplementing for the reasons listed

Protein powders, BCAA, and glutamine were strongly linked to performance reasons for supplementation; however, beta-alanine was not. Energy drinks were associated with muscle mass/strength, endurance, increase energy, and recovery but not overall athletic performance. Sport/electrolyte drinks, sport gels and gummies, sport/protein bars, and recovery drinks were associated with all performance reasons as was creatine, with the exception of increase energy (Table 4). Plant extracts were associated with muscle mass/strength, increase energy, overall athletic performance, however, the association was not as strong as with many other supplements (Table 4).

There were noticeable differences within the age groups in performance reasons for supplementation. The 11–13 year olds were the least likely to choose supplementation for performance reasons at 73 % and the 19–25 year olds the most likely at 94 % (*p* < 0.01; V = 0.144). Males had a higher use of supplements for performance reasons at 87 % as compared to females at 76 % (*p* < 0.01; V = 0.144). Level of competition did not influence supplement usage for performance-related reasons with all groups at approximately 80 %; however, aesthetic athletes were the least likely to use supplements for performance reasons at 64 % as compared to 77 % for intermittent athletes, 82 % for endurance athletes and 90 % for strength athletes (*p* < 0.01; V = 0.174).

### Supplement choice and influence of others

Reasons for supplementation under the purview of others included “because others (family/friends/teammates) do” and “someone told you to”. Although 44 % of athletes report others influenced their decision to use supplements, this category was the lowest ranking. Calcium and performance foods were associated with family/friends/teammates. Those athletes who reported taking supplements because “someone told them to” were also more likely to use multi-vitamin/multi-minerals, vitamin D, iron, calcium, fatty acids, and energy drinks (Table 5). Notably, supplement usage for reasons classified as due to the influence of others was 46 % in the 11–13 year olds and 50 % in the 14–16 year olds, whereas, it dropped to 35 % in 17–18 year olds and 30 % in 19–25 year olds (*p* < 0.01; V = 0.144). Gender, level of competition or sport type did not impact supplementation due to the influence of others.

**Table 5:** Associations between supplement use and influence-of-others reasons

Supplement	Family/Friends/Teammates (21 %)	Someone Told You To (35 %)
Multi-vitamin/Multi-mineral (67 %)	*χ* ^2^ = 1.074 *p* = 0.300 V = 0.044	***χ*** ^**2**^ **= 15.358** ***p*** **< 0.001** **V = 0.165**
Vitamin C (66 %)	*χ* ^2^ = 0.522 *p* = 0.470 V = 0.030	*χ* ^2^ = 1.273 *p* = 0.259 V = 0.048
B Vitamins (32 %)	*χ* ^2^ = 2.480 *p* = 0.115 V = 0.066	*χ* ^2^ = 1.255 *p* = 0.263 V = 0.047
Vitamin E (26 %)	*χ* ^2^ = 1.070 *p* = 0.301 V = 0.044	*χ* ^2^ = 2.727 *p* = 0.099 V = 0.070
Vitamin D (48 %)	*χ* ^2^ = 0.014 *p* = 0.904 V = 0.005	*χ* ^2^ = 9.449 *p* = 0.002 V = 0.130
Vitamin-enriched Water (65 %)	*χ* ^2^ = 2.108 *p* = 0.147 V = 0.061	*χ* ^2^ = 0.195 *p* = 0.659 V = 0.019
Iron (27 %)	*χ* ^2^ = 2.982 *p* = 0.084 V = 0.073	*χ* ^2^ = 4.773 *p* = 0.029 V = 0.092
Calcium (43 %)	*χ* ^2^ = 6.104 *p* = 0.013 V = 0.104	*χ* ^2^ = 7.059 *p* = 0.008 V = 0.112
Magnesium (17 %)	*χ* ^2^ = 3.587 *p* = 0.058 V = 0.080	*χ* ^2^ = 1.846 *p* = 0.174 V = 0.057
Protein Powder (51 %)	*χ* ^2^ = 0.005 *p* = 0.941 V = 0.003	*χ* ^2^ = 0.003 *p* = 0.959 V = 0.002
BCAA (8 %)	*χ* ^2^ = 0.051 *p* = 0.821 V = 0.010	*χ* ^2^ = 0.031 *p* = 0.859 V = 0.007
Beta-alanine (4 %)	*χ* ^2^ = 0.003 *p* = 0.957 V = 0.002	*χ* ^2^ = 0.012 *p* = 0.914 V = 0.005
Glutamine (8 %)	*χ* ^2^ = 1.393 *p* = 0.238 V = 0.050	*χ* ^2^ = 0.289 *p* = 0.591 V = 0.023
Fatty Acids (40 %)	*χ* ^2^ = 2.852 *p* = 0.091 V = 0.071	*χ* ^2^ = 7.947 *p* = 0.005 V = 0.119
Energy Drinks (27 %)	*χ* ^2^ = 3.643 *p* = 0.056 V = 0.080	*χ* ^2^ = 5.330 *P* = 0.021 V = 0.097
Sport/Electrolyte Drinks (90 %)	*χ* ^2^ = 5.129 *p* = 0.024 V = 0.095	*χ* ^2^ = 0.165 *p* = 0.685 V = 0.017
Sport Gels/Gummies (26 %)	*χ* ^2^ = 6.952 *p* = 0.008 V = 0.111	*χ* ^2^ = 0.882 *p* = 0.348 V = 0.039
Sport/Protein Bar (71 %)	*χ* ^2^ = 6.420 *p* = 0.011 V = 0.107	*χ* ^2^ = 3.154 *p* = 0.076 V = 0.075
Recovery Drinks (31 %)	*χ* ^2^ = 0.049 *p* = 0.826 V = 0.009	*χ* ^2^ = 0.365 *p* = 0.546 V = 0.025
Creatine (7 %)	*χ* ^2^ = 3.489 *p* = 0.062 V = 0.079	*χ* ^2^ = 0.012 *p* = 0.913 V = 0.005
Plant Extracts (47 %)	*χ* ^2^ = 0.376 *p* = 0.540 V = 0.026	*χ* ^2^ = 0.412 *p* = 0.521 V = 0.027

Statistically significant associations at *p* ≤ 0.001 are in bold. Percentages after the supplement type represent the percent of total athletes consuming the supplements listed. Percentages after the influence-of-others reasons represent the percent of total athletes supplementing for the reasons listed

## Discussion

The current study highlights the links between dietary supplement consumption and motivation for supplement use among young, Canadian athletes. These results are significant as they provide an, albeit indirect, assessment of the athletes’ knowledge of the benefits – real or purported – of dietary supplements as they relate to health and performance. Research in this demographic is particularly important, as the group consisted mostly of young, club level athletes who were less likely to have access to sport nutrition professional advisors, yet represent the majority of young athletes. Indeed, fewer than half had ever met with a dietitian and 38 % had previously attended a session on dietary or sport supplements [3]. Furthermore, the high prevalence of dietary supplement use suggests that research in this area is essential to ensure safe and effective supplementation practices.

Athlete self-evaluated perceived knowledge of the reasons for supplement use is generally low, although it does increase with age, whereby 60 % of athletes claim to know the reasons for all of the supplements they are consuming by ages 19–25. Indeed this purported level of knowledge is improved as compared to a 2003 study that found 10 % of Singaporean athletes, 85 % of which were 25 years or younger, self-rated their level of perceived knowledge regarding dietary supplements as good or excellent [9].

Vitamin and mineral supplements were associated with health-related reasons; however, evidence indicates these supplements are only effective in the case of a deficiency [10] suggesting a lack of congruency; with possible exceptions being vitamin D and calcium (females), which are reportedly low in athletes [11, 12]. In the case of micronutrient deficiencies, dietary changes rather than supplementation should be the primary target to bring intakes into the healthy range. The consumption of sport bars, protein powders, and amino acid supplements was linked to reasons for choosing supplements associated with improved health, particularly immune system enhancement and to improve diet quality. In cases of inadequate dietary intake of protein, performance foods high in protein (protein bars, recovery drinks, protein powders, etc.) would be beneficial, suggesting congruence between the actions and motivation; however, when dietitians observed nutritional habits in young athletes, they found protein intakes to be more than sufficient. Average protein intake in male athletes was 2.3 g/kg body weight, which is significantly higher than the recommended upper intake for athletes of 1.7 g/kg body weight, and 86 % of females met the recommended intake with an average intake of 1.4 g/kg body weight [13]. Furthermore, in Canadian, high-performance athletes, protein intakes were 1.7 g/kg body weight from food alone and 1.8 g/kg body weight from food and supplements in females and 1.9 g/kg body weight from food alone and 2.1 g/kg body weight from food and supplements for males [14], clearly indicating protein is not a nutrient of concern for most athletes.

Evidence of congruence between health-related reasons for supplementation and actual practice also exists in that creatine, energy drinks, sport gels and gummies, and sport drinks were rarely associated with health reasons; indicating athletes are potentially able to distinguish between supplementation for health and performance in these cases. Conversely, it has been suggested that consuming a 6 % or greater carbohydrate beverage, such as a commercial sport drink, during prolonged exercise can reduce the risk of upper respiratory tract infections, potentially creating the case for sport drinks and improved immune function [15]. Fatty acid consumption was associated with health-related reasons and although omega-3 fatty acids are believed to have anti-inflammatory properties, there is currently insufficient evidence to determine if they are capable of altering immune function in athletes [15]. Plant extracts encompass a large group of dietary supplements, however, echinacea and ginseng are consistently popular [16] and these examples were provided in our questionnaire. With respect to motivation for supplementation with plant extracts, health-related reasons such as “prevent or heal illnesses or injuries”, “support immune system” and “strengthen overall health” [16] have been reported, which is in agreement with our participants. Unfortunately, there is a paucity of high-quality, human studies to evaluate the effects of herbal supplements on health-related reasons for supplementation in athletes and as such, the potential benefits cannot be confirmed [16]. While the effectiveness of most plant-based natural health products remains to be determined, one should acknowledge these products are generally marketed for health reasons.

The use of dietary supplements to enhance performance in young athletes is controversial. Some experts recommend that athletes under the age of 18 years avoid using dietary supplements unless required for a medical reason and only in cases where they are supervised by a professional [17]. Although this advice is sound in that it errs on the side of safety, it is perhaps not realistic and certainly the high rates of dietary supplement use suggest athletes are not heeding this conservative message. Regardless, if an athlete opts to take supplements, the chosen dietary supplements should be found to be effective in relation to their performance goals.

A large percentage of athletes consumed vitamin or mineral supplements and there was an association with motivation for enhanced performance; however, as with health-related reasons, the conventional thinking is that vitamin and mineral supplements will not improve performance, except in the case of a deficiency, illness or restricted food intake [10]. Vitamin-enriched waters were also associated with performance reasons, however, are unlikely to benefit athletes if dietary intakes are adequate and concerns have been raised regarding the added sugars in some vitamin-enriched water options. Protein powders and various amino acid products were commonly reported in athletes looking to improve performance, a finding supported by others [7, 18]. These products are frequently advertised to increase muscle mass and performance [19], indicating some level of congruence between the performance goals and supplement choice. Protein supplementation has been found to improve muscle hypertrophy and strength, as well as aerobic and anaerobic power, when combined with appropriate training [20], conversely, less support exists for potential benefits with recovery of muscle function, muscle soreness, and muscle damage [19].

Energy drink consumption was also positively associated with performance reasons, notably “increase energy” and “muscle mass/strength” in these athletes. Energy drinks may improve neuromuscular performance, delay central nervous system fatigue, and increase endurance performance [21, 22], however, the optimal dose and their potential superiority to a sport drink is unclear. Furthermore, there are concerns with energy drinks as a hydration choice, in those who are not habituated, as they may increase water and sodium losses and have a mild thermogenic effect [21]. Other potential side effects include: increased sugar intakes, gastrointestinal problems, tachycardia, anxiety, headaches and insomnia [21, 22]. In general, energy drinks are contraindicated for those under the age of 18 years and their consumption should be discouraged except in very specific circumstances and under parental guidance [22–24]. Creatine supplementation was associated with “muscle mass/strength”, “endurance”, and “overall athletic performance”; indeed there is evidence to suggest creatine may be efficacious in enhancing muscle hypertrophy and performance in short, high-intensity activities, however, the majority of the research has been conducted in adult males [25]. Younger athletes could benefit from creatine supplementation, as their ability to regenerate high energy phosphates is reduced; nonetheless, the International Society of Sports Nutrition only deems creatine supplementation acceptable if the athlete is past puberty, competing at a high level, has parental approval, is supervised by a qualified professional, and uses an appropriate dose [25, 26].

Consuming carbohydrates or carbohydrate-electrolyte combinations during exercise can delay fatigue, thereby improving performance in intermittent and prolonged endurance exercise [27, 28]. Arguably, congruence exists between the use of sport/electrolyte drinks and sport gummies/gels and performance-based motivations for supplement use. Carbohydrate intake during exercise has been identified as an area where young athletes do not meet the recommendation of 30–60 g/h, in at least one study [13], suggesting that this link could be highlighted in sport nutrition education. The use of these products should be carefully considered in the context of the duration and intensity of the exercise, however, as there is a concern with the use of sport drinks and excess calories in the form of added sugars [24]. Plant extracts were also associated with performance reasons, yet controversy exists regarding the potential benefits of common plant extracts such as echinacea [16, 29, 30] and ginseng [31] on athletic performance.

Vitamin and mineral supplements, fatty acids, energy drinks, sport/protein bars, and gels and gummies were associated with “because someone told you to” or “because family/friends/teammates do”. These results are in-line with a study in young, elite German athletes that found coaches (37 %), family (30 %), physicians (29 %), and nutritionists (14 %) provided information on dietary supplements. Furthermore, 16 % of athletes learned about dietary supplements from the media and 27 % reported that they did not receive any information about dietary supplements from anyone [32]. As may be expected, the younger the athlete, the more likely they are to consume supplements because others have told them to. Certainly, encouraging young athletes to heed the advice of others, particularly their parents or guardians, is advisable; however, the sport nutrition knowledge of these individuals is uncertain. Influence of others on dietary supplement use, particularly those who may not possess the appropriate knowledge, is a common cause for concern in athlete populations [33, 34]. Clearly education is required not only for the athletes but also their support network, as there is obviously a willingness to try supplements based on the recommendations of others rather than personal knowledge.

Our study is limited in that young participants completed the questionnaire using personal recall and there may be errors due to a lack of understanding. Delivering the questionnaire in a “face-to-face” mode, where they could ask questions and receive explanations minimized this limitation. Additionally, the athletes were not specifically asked the reason for consuming each supplement; rather, the analysis looks at associations within the data, therefore, the magnitude of the association was reported and should be considered in the interpretation of the results. Future research that directly measures athlete knowledge regarding dietary supplements and relates this knowledge to their motivation for supplementation and actual intakes would be valuable to gain a more in-depth understanding.

## Conclusions

Young athletes are consuming dietary supplements for reasons related to health, performance, and under the recommendation of others. Although in some cases supplement choices are congruent with the established benefits of the supplement, there are many instances where there is a lack of evidence to support supplementation practices as they relate to their self-reported motivations for supplement use. Additional research into the safety and effectiveness of dietary supplements in this demographic is required. Furthermore, evidence-informed educational programs are required for the athletes and their support personnel to ensure supplements are being used efficaciously to promote health and performance. Current sources of reliable information include on-line resources developed and monitored by sporting organizations or nutrition associations (i.e Coaching Association of Canada coach.ca) and Registered Dietitians who provide training in nutrition and supplement use. A second recommendation would be for sporting organizations to facilitate nutrition sessions for their athlete support personnel, who could then inform the parents and athletes. Future, studies should assess the effectiveness of a variety of educational interventions and their impact on nutrition knowledge and dietary habits and supplement use.
